# Etymologia: Methylotroph

**DOI:** 10.3201/eid2203.ET2203

**Published:** 2016-03

**Keywords:** etymologia, methylotroph, bacteria, methanol, methane, single-carbon compounds

## Methylotroph [methʹil-o-trofʺ]

From the Greek, *methy*, “wine,” plus *trophē*, “food,” methylotrophs (Figure) are a diverse group of bacteria that can synthesize all their cell constituents from reduced single-carbon compounds, such as methanol or methane, or multicarbon compounds with no carbon–carbon bonds. The first methylotroph, *Methylomonas methanica*, was described (as *Bacillus methanicus*) grown aerobically on methane by Söhngen in 1906.

**Figure:**
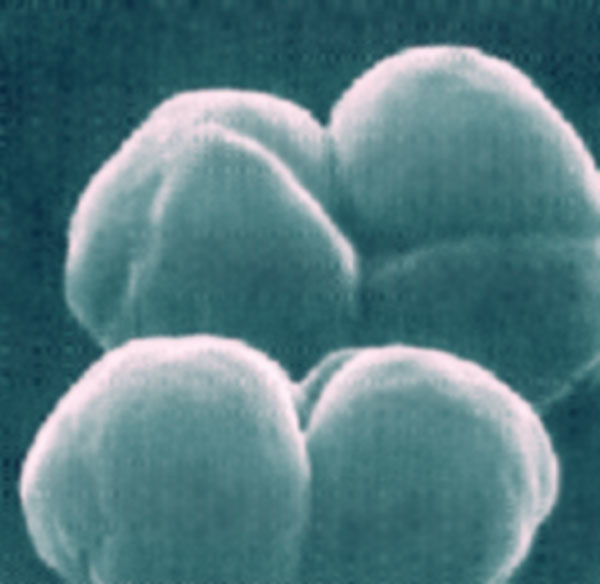
Methylomonas methanica
